# Latent negative precipitation for the delineation of a zero-precipitation area in spatial interpolations

**DOI:** 10.1038/s41598-021-99888-4

**Published:** 2021-10-14

**Authors:** Taesam Lee, Ju-young Shin

**Affiliations:** 1 Department of Civil Engineering, ERI, Gyeongsang National University, 501 Jinju-daero, Jinju, Gyeongnam, 660-701 South Korea; 2 High-Impact Research Department, National Institute of Meteorological Sciences, Jeju, South Korea

**Keywords:** Climate sciences, Environmental sciences, Hydrology, Natural hazards

## Abstract

The spatial interpolation of precipitation has been employed in a number of fields, including by spatially downscaling the Global Circulation Model (GCM) to a finer scale. Most precipitation events become more sporadic when the coverage area increases (i.e., a portion of the points experience zero precipitation). However, spatial interpolations of precipitation generally ignore these dry areas, and the interpolated grids are filled with certain precipitation amounts. Subsequently, no delineation of dry and wet regions can be made. Therefore, the current study suggested a novel approach to determine dry areas in spatial interpolations of precipitation events by assigning latent negative precipitation (LNP) to points with observed precipitation values of zero. The LNP-assigned points are then employed in a spatial interpolation. After that, the dry region can be determined using the negative region (i.e., points with zero precipitation). The magnitude of LNP can be defined by multiplying the precipitation values of neighboring stations by a tuning parameter. The LNP method and the tuning parameter are tested on weather stations covering South Korea. The results indicate that the proposed LNP method can be suitable for the spatial interpolation of precipitation events by delineating dry and wet regions. Additionally, the tuning parameter plays a special role in that it increases in value with longer precipitation durations and denser networks. A value of 0.5–1.5 can be suggested for the tuning parameter as a rule of thumb when high accuracy for final products of interpolated precipitation is not critical. For future studies, the LNP model derived herein can be tested over much larger areas, such as the United States, and the model can also be easily adopted for other variables with spatially sporadic values.

The spatial interpolation of precipitation plays a critical role when mapping sparse point precipitation data from weather stations to obtain input data for distributed hydrologic models such as Vflo^1,2^, evaluating weather prediction models^3^, and assessing the impacts of climate change on local watersheds through spatial downscaling^4–7^. A number of spatial interpolation schemes have been proposed in the literature^8^, such as Thiessen polygons^9^, inverse distance weighting (IDW)^10^, linear (and nonlinear) spatial interpolations^11^, and geostatistical kriging^12,13^.

In recent years, these spatial interpolation techniques have been popularly used to statistically downscale hydrometeorological variables when conducting climate change assessments^14–20^. A number of statistical downscaling methods have been used to simulate precipitation data for hydrologic and agricultural model inputs^18,20–22^, such as bias correction spatial downscaling^23,24^, constructed analogs^6^, and conditional simulations^25^.

In implementing a spatial interpolation of precipitation data, a portion of the observed points (or grids) often have observed precipitation values of zero. All target interpolating grids are filled with a certain amount of precipitation in most spatial interpolation methods, such as IDW, linear spatial interpolation, and kriging, unless all the considered stations have precipitation values of zero. The interpolated precipitation thus results in a high frequency of low precipitation values. These results might not be favorable for applications in water resource management and distributed hydrologic models since they reduce dry regions and increase outflows.

Therefore, a novel approach for spatially interpolating precipitation regarding the determination of wet and dry regions is proposed in the current study. The applicability of the proposed model was tested using weather stations in South Korea.

## Mathematical Background

### Spatial Interpolation

IDW is one of the most common spatial interpolation approaches and involves assigning weights to the observed values of neighboring stations according to the distances between the neighboring stations and the target point and taking weighted averages. The precipitation, $\widehat{P}$, for a target point or grid can be calculated with IDW, when *P*_*s*_, *s* = 1,…,*S,* where *S* is the number of neighboring stations, as follows:

1 $$ \hat{P} = \frac{{\mathop \sum \nolimits_{s = 1}^{S} P_{s} w_{s} }}{{\mathop \sum \nolimits_{s = 1}^{S} w_{s} }} $$

where the weights are *w*_*s*_ = 1/*d*_*s*_^2^ and *d*_*s*_ is the distance between the target point and station *s*. IDW is a simple and efficient interpolation method^26^, and more complex methods such as multiple linear regressions and kriging interpolation can perform better but require sufficient data densities^27^.

The simplest approach for the spatial interpolation of rainfall is to assign the same value to the target point as that of the closest station, called the nearest-neighbor (NN) method, such that the following expression is true.

2 $$ \hat{P} = \min (d_{s} ,s \in \left\{ {1,...,S} \right\})P_{s} $$

Note that if precipitation is interpolated with this NN method, especially in gridded cases, Thiessen polygons can be constructed^28^.

### Proposed model description

To spatially interpolate precipitation, including precipitation in wet regions (i.e., precipitation > 0) and dry regions (i.e., precipitation = 0), an appropriate method must be proposed. To the best of the authors’ knowledge, no appropriate method exists. In the current study, the latent negative precipitation (LNP) method is proposed. First, a negative precipitation amount is assigned before interpolation to stations with precipitation values of zero with the same magnitude as that of a precipitated neighbor station. This negative value is latent since it is not measured, but it indicates how much driving force is required to make a neighboring station dry.

**Figure 1:**
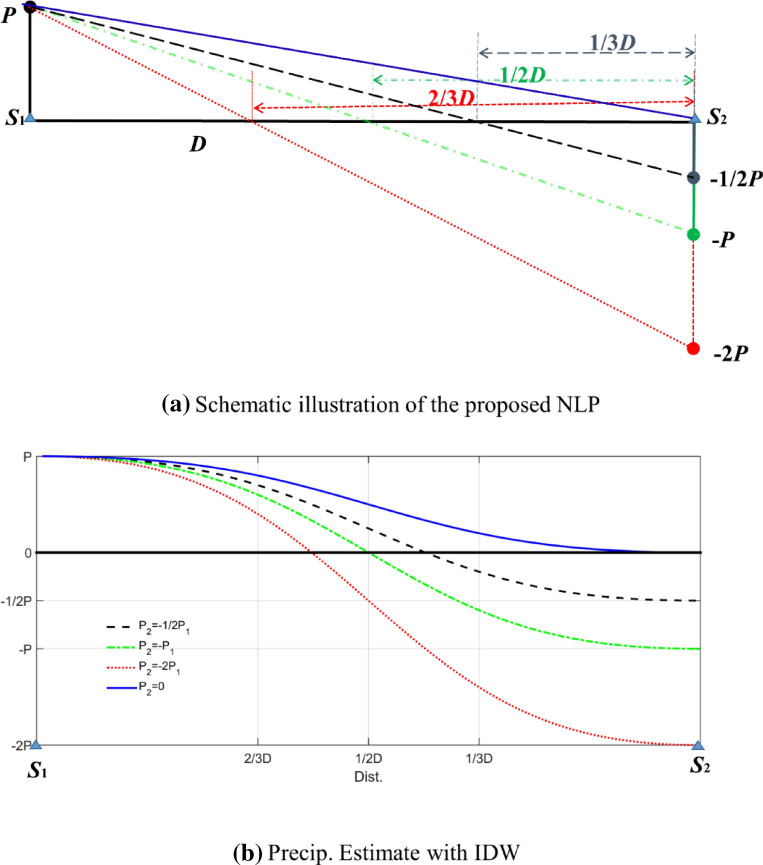
Schematic illustration of negative latent precipitation (**a**) and spatial interpolation performed with IDW for LNP (**b**). Note that the left side point (*S*_1_) indicates a weather station that records a precipitation amount of *P*, while the weather station on the right side (*S*_2_) records no precipitation. Three different negative values, −2*P*, −*P*, and 1/2*P,* are assigned instead of a value of zero as latent precipitation. For example, when −2*P* is assigned, the 2/3*D* area among *D* is eventually a zero-precipitation area, as shown in the top panel (**a**). The precipitation estimates obtained with IDW in the bottom panel (**b**) were calculated using the simple equation *P*_*IDW*_ = (*a*^2^ + *b*)/(*a*^2^ + 1)*P,* where *a* = *D*_2_/*D*_1_ and *b* = *P*_2_/*P*_1_. Note that *a* and *b* are the ratios of the distance and precipitation, respectively, for site 1 and site 2.

The physical justification is the following. When a station has a high precipitation value, often accompanied by low pressure, a neighboring station that is dry may be affected by strong high pressure based on meso and macro scales. The detailed extensive description of the LNP method is explained with the diagram in Fig. 1.

A case with two weather stations is shown in Fig. 1. Station *S*_1_ on the left side has a *P* amount of rain, while station *S*_2_ on the right side has no precipitation. For the spatial interpolation of precipitation with two stations separated by a distance of *D*, the interpolation values between the two stations can be linearly interpolated, as shown by the solid blue line in the top panel (a), or interpolated with IDW, as shown in the bottom panel (b). The interpolation results have no zero-precipitation areas except at station *S*_2_. This drawback can be problematic when interpolated data are used for drought analyses, water resource management, or flood analyses. In recent decades, the spatial downscaling of global climate model (GCM) outputs has been popularly employed with spatial interpolation techniques^4,5^ requiring adequate delineations of zero-precipitation areas.

Therefore, a special remedy that allows spatial interpolations with the delineation of zero-precipitation areas is proposed in the current study. The dry severity of these regions is as high as the precipitation amount of the neighboring weather stations since the wet weather system is blocked by the dry system and a severely wet system must be blocked by a severely dry system to have an area with zero rainfall. Therefore, it is suggested that a dry weather system can be represented with LNP for stations with zero precipitation.

The procedure can be summarized as follows: (1) LNP is assigned to the stations with zero precipitation; (2) the target area is spatially interpolated with LNP values and other nonzero precipitation values; and (3) the negative-value area is set as a zero-rainfall area. In this procedure, the dry region is defined according to the strength of LNP. For example, when LNP is equal to -2*P*, two-thirds of the area (presented with *D*) has a value of zero in the linear interpolation, as shown in the top panel (a) of Fig. 1, and less than two-thirds of area (D) has a value of zero in IDW, as shown in the bottom panel (b); the situation is reversed in the case of -1/2P.

The severity of dryness represented with LNP can differ with each storm event and area. Even though the LNP severity of each storm event cannot be modeled, the overall regional effect can be considered using a tuning parameter (i.e., represented with -1/2 ~ -2 in Fig. 1). This parameterization is discussed following a detailed description of spatial interpolation using LNP.

Assuming that *M* weather stations have precipitation values of zero among *S*_*i*_, (*i* = 1,…,*S* where *S* is the number of neighboring stations), the weather stations with zero precipitation are described as *S*_m_^*^ (*m* = 1,…,*M*). The objective is to spatially interpolate a certain gridded area (say *N*_*L*_ = *N*_*C*_ × *N*_*R*_ where *N*_*C*_ and *N*_*R*_ are the numbers of columns and rows, respectively) containing the considered weather stations *S*_*i*_, (*i* = 1,…,*S*) with some zeros. The detailed procedure is as follows for weather stations *S*_*i*_, *i* = 1,…, *S*.

1. Calculate the distances between stations and indicate the stations that have zero precipitation. Here, assume M stations have zero precipitation.
2. Estimate LNP for the stations (*S*_m_^*^; *m* = 1,…,*M*) with zero precipitation using spatial interpolation techniques such as NN or IDW.
  3 $$ \hat{P}_{m}^{*} = \frac{{\mathop \sum \nolimits_{j = 1}^{S - M} P_{j} \left( {1/d_{j}^{2} } \right)}}{{\mathop \sum \nolimits_{j = 1}^{S - M} 1/d_{j}^{2} }}\;\;{\text{for}}\;{\text{IDW}} $$
  4 $$ \hat{P}_{m}^{*} = \min \left( {d_{j} ,j \in \left\{ {1,...,S - M} \right\}} \right)P_{j} \;\;{\text{for}}\;{\text{NN}} $$
  5 $$ LNP_{m} = - \hat{P}_{m}^{*} $$
3. Spatially interpolate the target gridded area with the observed precipitation values, *P*_*j*_ (*j* = 1,…,*S*-*M*), and the estimated *LNP*_*m*_ (*m* = 1,…,*M*). Let the interpolated precipitation values be represented as $\tilde{P}_{l}$ (*l* = 1,…, *N*_*L*_; the number of grids).
4. Set the negative values as zero for the spatially interpolated grids, as shown below.
  6 $$ \hat{P}_{l} = \left\{ {\begin{array}{ll} {\tilde{P}_{l} } \hfill &\quad {{\text{if}}\;\tilde{P}_{l} > 0} \hfill \\ 0 \hfill &\quad {{\text{otherwise}}} \hfill \\ \end{array} } \right. $$

In Fig. 1, weather station *S*_1_ on the left side has *P* amount of rain, while *S*_2_ has dry conditions. By assigning the negative value of *S*_1_ as –P to the dry site (*S*_2_), half of the distance (1/2*D*) is negative by linear interpolation (the dash-dotted green line in Fig. 1). By setting the negative area to zero, this area becomes a nonprecipitated area. Note that the NN method results in the same 1/2*D* area.

However, the dry area can differ when assigning different LNP values. For example, an LNP value of −2*P* enforces the zero-precipitation area as 2/3*D* (see the dotted red line in Fig. 1), while a value of -1/2*P* enforces the zero-precipitation area as 1/3*D* (the dashed black line in Fig. 1). Even though an appropriate LNP value length can differ among each precipitation event, overall adjustment can be made by parameterizing the proportion as follows:

7 $$ LNP_{m} = - \lambda \hat{P}_{m}^{*} $$

where *λ* plays the role of a tuning parameter.

#### Estimation of the tuning parameter

To estimate the tuning parameter *λ*, *K*-fold cross validation (KFCV) was employed. With the presumed tuning parameter *λ*, KFCV can be performed by the following procedure: (1) dividing the dataset into *k* subsets, (2) fitting a model with the data excluding each subset, (3) predicting the excluded subset and calculating the cross-validation errors, and (4) repeating this *K* times. The cross-validation error is calculated as follows:

8 $$ E_{K} \left( \lambda \right) = \mathop \sum \limits_{k = 1}^{K} \mathop \sum \limits_{i = 1}^{{n_{k} }} \left\{ {P_{i} - \hat{P}_{i}^{ - k} \left( \lambda \right)} \right\}^{2} $$

where $\hat{P}_{i}^{ - k} \left( \lambda \right)$ is the predicted precipitation with the LNP interpolation method and the data excluding each *k*th subset and *n*_*k*_ is the number of data points in the *k*th subset. The tuning parameter *λ* is taken as the minimum value of $E_{K} \left( \lambda \right)$. Note that the fitting model includes spatial interpolation models such as NN and IDW, including the LNP for zero-precipitation areas as suggested. Also, root-mean-square-error (RMSE) was estimated to show the performance of the proposed LNP model and is denoted as:

9 $$ RMSE = \sqrt {1/n\mathop \sum \limits_{i = 1}^{n} \left\{ {P_{i} - \hat{P}_{i} } \right\}^{2} } $$

where $\hat{P}_{i}$ is the predicted precipitation.

In this study, ten iterations were adopted for KFCV. The first to ninth iterations had seven stations, and the tenth iteration had ten stations. Eight durations, 1, 2, 3, 4, 6, 8, 12, and 24 h, were employed for the precipitation event duration. To investigate the impacts of the spatial coverage of a precipitation event on the interpolation methods, various numbers of wet stations, such as 10, 20, 30, 40, 50, and 60, were selected, for which the precipitation depths were higher than 0 mm. For the case of ten wet stations, any precipitation event with more than ten wet stations during the event was defined as a precipitation event. In addition, precipitation events were omitted in the cross-validation if the total depth of precipitation for all employed stations was lower than 10 mm to exclude very light precipitation events and to attenuate errors in the observations.

### Study area

**Figure 2:**
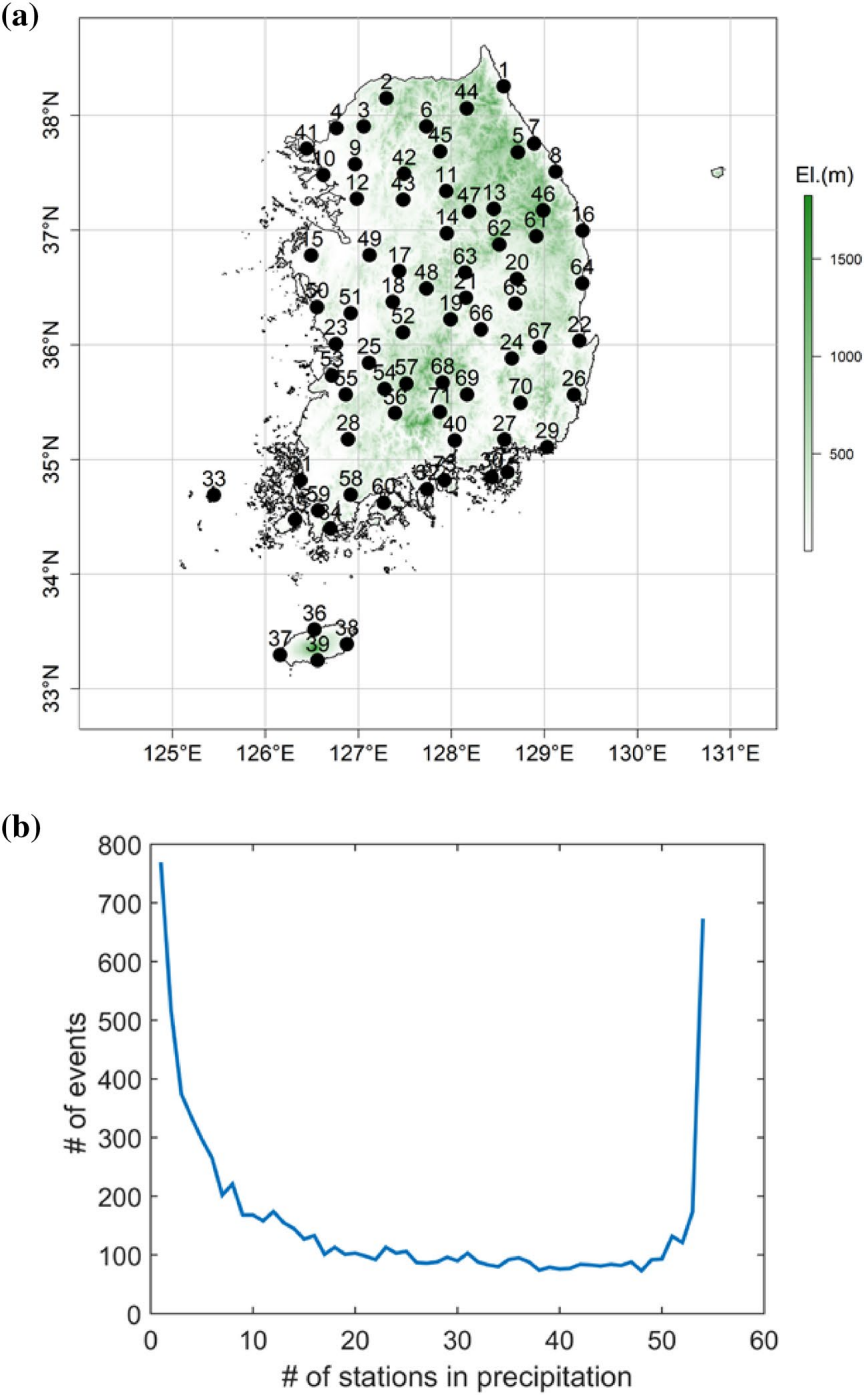
(**a**) Locations of weather stations employed and (**b**) number of events corresponding to the number of stations with precipitation. For example, the number of events in which just one station recorded precipitation is approximately 750 among 73 stations in South Korea.

To validate the suggested model, 73 stations over South Korea were applied; these stations are run by the Korea Meteorological Agency (KMA) and are shown in Fig. 2a and Table S1 of the Supplementary Material for their latitude and longitude. South Korea illustrates serious rainfall deviation, as most of the annual rainfall falls during the rainy season, leading to vulnerability to floods. Extreme rainfall events also often occur from tropical cyclones or severe thunderstorms, and events are reportedly increasing^29^. Assessing future extreme rainfall events is critical, and the spatial downscaling of GCM outputs is crucial since the resulting areas affected by rainfall events are too small to obtain by directly applying GCM outputs.

Figure 2b presents the number of studied precipitation events along with the number of precipitated stations. The frequency indicates that a large number of partial precipitation events occur. For example, precipitation events with only one station occurred approximately 750 times over the study period, while 10–50 stations experience approximately 100 precipitation events each. Additionally, events in which all the stations recorded precipitation occurred approximately 700 times.

## Results

**Figure 3:**
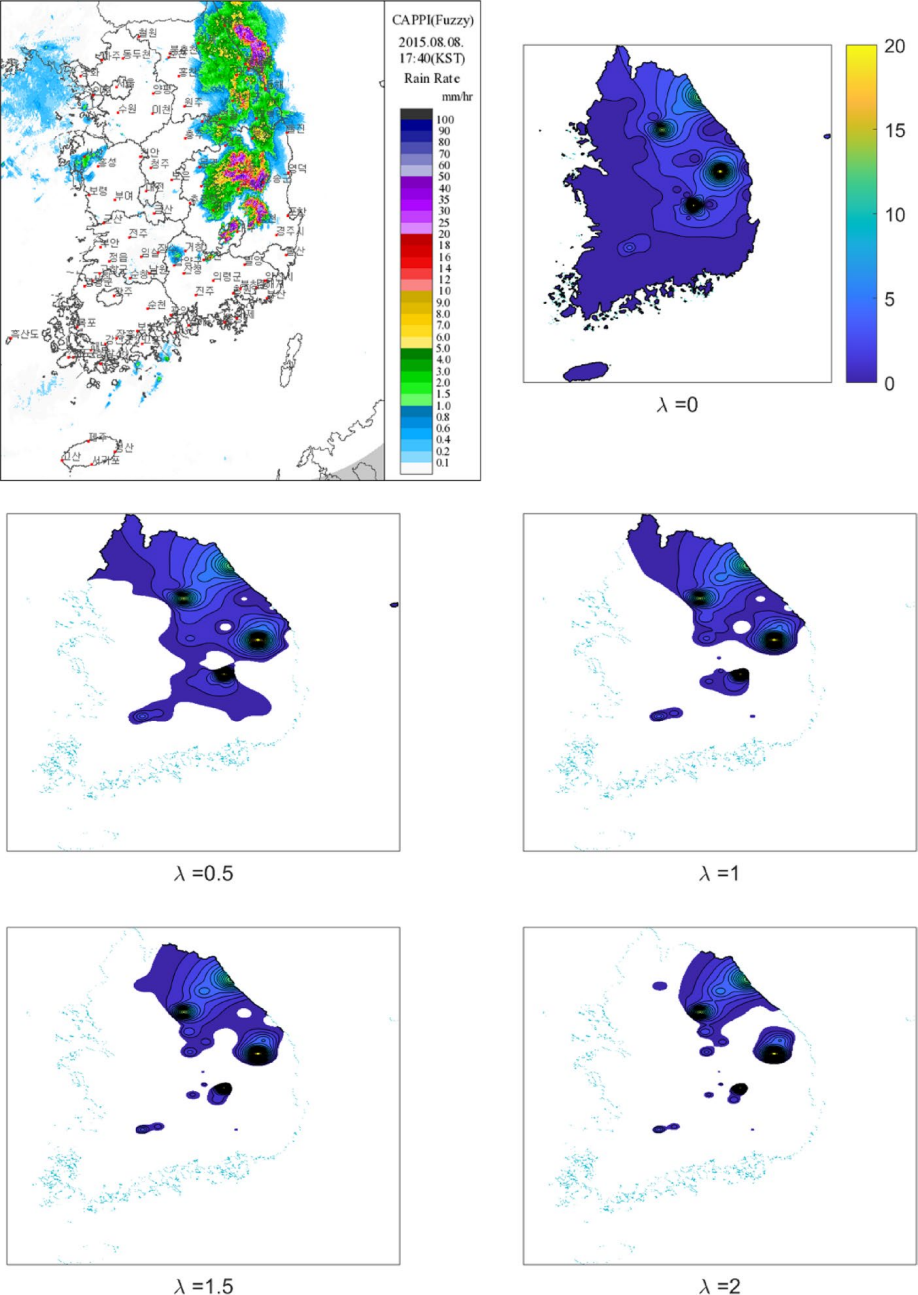
Example of the radar image for the precipitation (mm/hr) on August 8, 2015 at 17:40 (top left panel) and the proposed spatial interpolation LNP and IDW technique with different lambdas. Note that the estimated optimal value of lambda is 0.51 for this case. The figures were created by MATLAB 2021a (https://www.mathworks.com/products/matlab.html).

Figure 3 illustrates one of the precipitation events that occurred over South Korea by interpolating the country with IDW and enhancing the LNP method to indicate dry regions. The top left panel of the figure presents the constant altitude plan position indicator (CAPPI) of the radar product provided by KMA for August 8, 2015, at 17:40 (downloaded from the site: https://www.kma.go.kr/eng/weather/images/radar.jsp), and the other panels of the figure illustrate the interpolated precipitation obtained using the rainfall that occurred during 17:00–18:00 on August 8, 2015 as measured by the 73 weather stations with different lambda values. Note that (1) CAPPI products do not directly indicate the amount of precipitation because they measure the amount of water vapor present before it falls to the ground, and the time interval is different from that of the right side of the figure; (2) the estimated optimal *λ* for this case was 0.51 that are close to the one of the left and second-row panel of the figure. The spatial rainfall distributions of the radar and the one close to the optimal one (the left and second-row panel) coincides well with each other. The dry area over South Korea is also represented well with the LNP model. The top right panel presents the case when *λ* = 0, and no dry area can be shown since the LNP method was not adopted. In contrast, the dry area increases as λ increases as shown in the second and third-row panels. This indicates that *λ* must be adequately estimated to be used effectively and illustrates that the proposed LNP model can be acceptable for use in spatial interpolations, especially regarding the delineation of dry regions.

**Figure 4:**
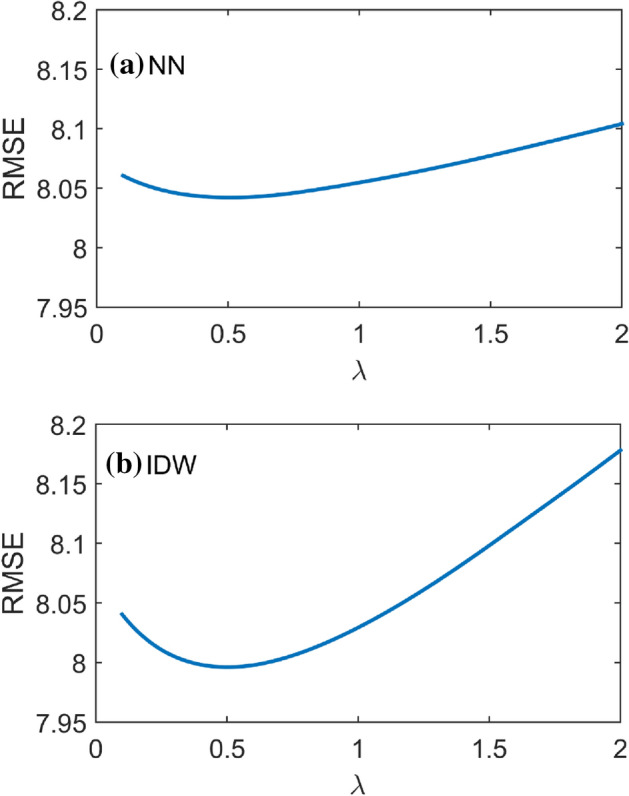
RMSEs of k-fold cross-validation outputs obtained with nearest-neighbor (NN) and inverse distance weight (IDW) interpolations for the LNP method when estimating the best λ.

In Fig. 4, the optimal *λ* value was found by assigning LNP amounts obtained with IDW and NN, as in Eq. (3) and Eq. (4). Both cases show a similar optimal value of *λ* = 0.5. However, IDW shows a lower RMSE than NN, indicating that assigning LNP using the weighted averages of neighboring stations according to inverse distances (i.e., IDW) might be better than using nearest-neighbor estimations (see Table 1). By estimating the lambda value, a precipitation event can be interpolated by separating the wet and dry regions, as shown in Fig. 3.

**Table 1:** RMSEs of the estimated precipitation with nearest-neighbor (NN) and inverse distance weights for different lambda values.

lambda	NN	IDW
0.0	8.343	8.343
0.5	8.042	7.996
1.0	8.055	8.029
1.5	8.077	8.098
2.0	8.104	8.178
2.5	8.133	8.263
3.0	8.162	8.351

Further experiment and test were made to provide the statistical test especially for comparing the interpolation with and without the LNP model. In the experiment, 60 stations were employed to interpolate while the other 13 stations were assumed not to have observed precipitation data. The precipitation at 13 stations was interpolated using IDW with and without the LNP model. The interpolated precipitation data are used for the Kolmogorov–Smirnov test (KS test) with observed precipitation data. KS test is a nonparametric test that can be used to compare two samples or a sample with a reference probability distribution (e.g. observed distribution). Smaller of KS statistic between the distribution from the interpolated data and the one from the observed data indicate better agreement with the observed data. The KS test statistics for two interpolated data sets (i.e. with and without the LNP model) are presented in Table S2 of the Supplementary Material. The precipitation data interpolated with LNP provide a smaller KS value than the other. The interpolated data with LNP can provide a more robust outcome than without the LNP model. For visual inspection, the histograms of observed and interpolated precipitation for three durations are presented in Figure S1 of the Supplementary Material. As shown in this figure, the histogram of precipitation with LNP is more similar to the histogram of observed precipitation than the one without the LNP model.

The effects of the density of the weather station network and precipitation duration on the tuning parameter were further analyzed. The density of the network can substantially affect the tuning parameter because a longer interpolation area range must be estimated with a lower density network. To test this characteristic, an experiment was performed by randomly selecting stations with varying network densities from 40 to 100 km/station. A further range was not feasible due to the size of the tested area. Using KFCV, the optimal values were estimated and averaged. The detailed results of each case are presented in Figs. S2–S7 of the Supplementary Material. Additionally, the effects of different precipitation durations on the tuning parameter (*λ*) were also tested.

**Figure 5:**
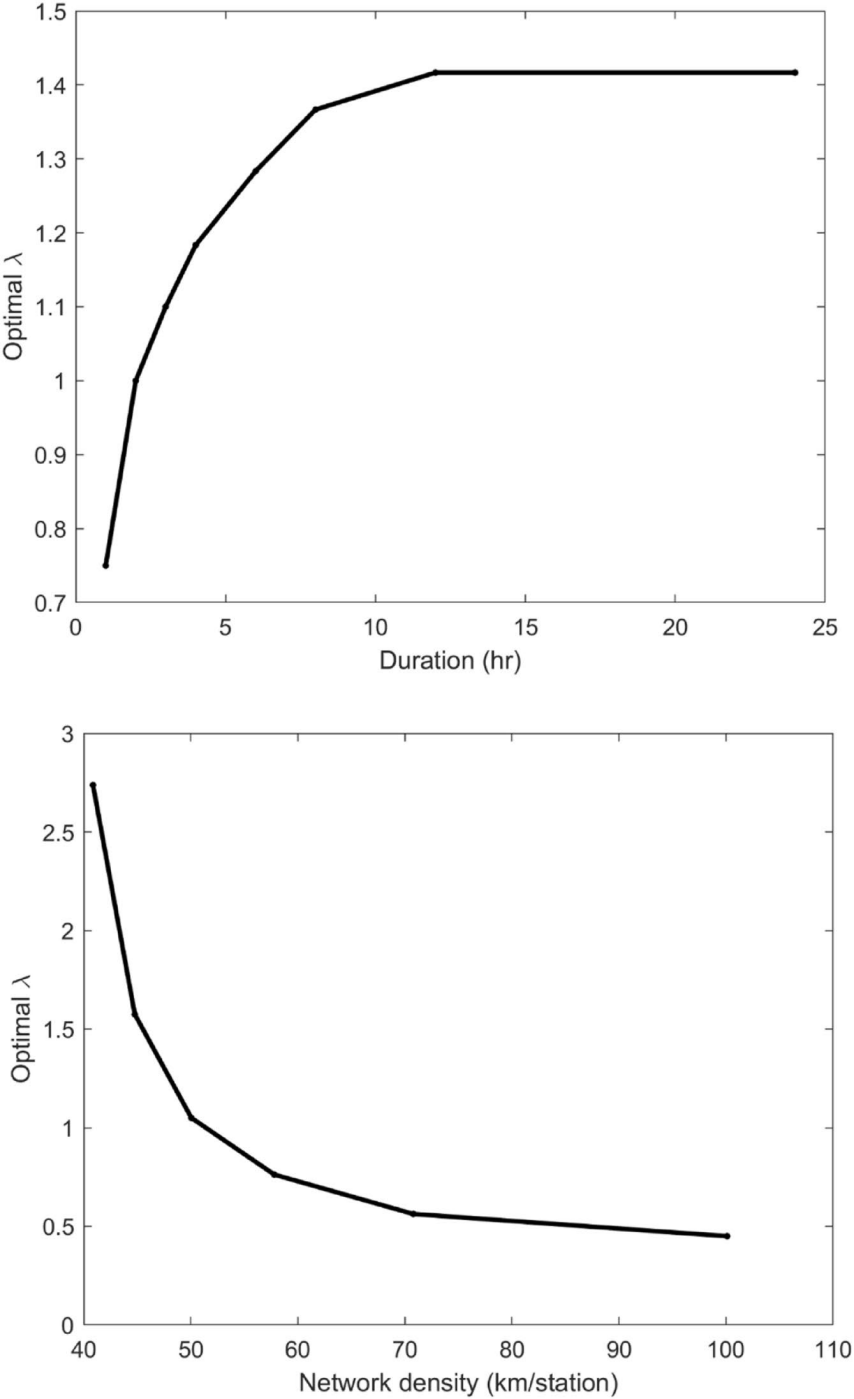
Variation in the optimal lambda along with the precipitation duration (top panel) and network density (bottom panel). Note that the average distance to the nearest station in the current study is 30 km.

The characteristics of the tuning parameter as affected by the density network are well presented in the bottom panel of Fig. 5. A network density higher than 50 km/station (i.e., each station has an average distance of 50 km from another station) presents a tuning parameter value of less than one, and the parameter value converges to 0.5 with increasing distance. With a smaller network density, the optimal tuning parameter presents a higher value. This result might be induced by the fact that more accurate interpolated precipitation outputs are possible with a denser network. This intensifies the magnitude of the tuning parameter since dry regions are well-established in high-density networks.

The estimated tuning parameter increases up to 1.4 as the precipitation duration increases, as shown in the top panel of Fig. 5. Note that a precipitation event with a longer duration is likely to have a larger wet area than one with a shorter duration since the area is exposed longer to the storm system. In this case, wet areas in precipitation events with longer durations illustrate less sparsity than wet areas in shorter precipitation events. To obtain a small dry area in a large wet area, a high optimal parameter value is needed.

It is obvious that interpolated precipitation estimates in wet areas will decrease compared with the case that the LNP approach is not applied because the LNP approach assigns a negative value (but eventually zero values) to the stations with no precipitation. In order to investigate this behavior further, the average of the mean areal precipitation for all rainfall events using IWD with and without the LNP during the studied period over South Korea was calculated and presented in Table 2. As shown in Table 2, the IDW with the LNP is smaller than IDF without the LNP as assumed. The difference between rainfall amounts from IDW with and without the LNP is around 0.45 mm for all employed durations and about 25 to 7 percent. The difference increases as the duration become longer. For rainfall events of 1- and 24-h duration, the difference of rainfall amount is 0.26 mm and 0.55 mm, respectively.

**Table 2:** Average of mean areal precipitation for all rainfall events during the studied period from 2003 to 2017 across South Korea. Note that RD = (IDW w/o LNP—IDW w LNP)/(IDW w/o LNP)*100.

Duration (hr)	IDW without LNP (mm)	IDW with LNP (mm)	RD(%)
1	1.071	0.809	24.463
2	1.656	1.292	21.981
3	2.149	1.729	19.544
4	2.569	2.121	17.439
6	3.392	2.887	14.888
8	3.987	3.463	13.143
12	5.22	4.667	10.594
24	7.603	7.075	6.945

With the overall assessment of the tuning parameter, it is concluded that the parameter has a critical role in determining dry areas via spatial interpolation. The parameter increases as the duration of a precipitation event increases and as the network density of weather stations increases. In a general case, the tuning parameter value can be set between 0.5 and 1.5 as a rule of thumb if the accuracy of the interpolated area is not significantly important.

The physical assumption of the delineation of the dry area due to pressure difference was thoroughly tested. Mean sea level (MSL) pressure data from ERA5 (5th Generation ECMWF atmospheric ReAnalysis of the global climate) was used to calculate the pressure at the points of interest. The MSL pressure near Korean peninsula during precipitation events in 2016 was matched to the grid points of precipitation data using IDW, and then characteristics of MSL pressure for dry and wet points were estimated. Figure S8 presents the spatial distribution of MSL pressure and precipitation data on April 4, 2016. There is a good agreement between pressure and wet/dry area.

In the current study, GPM (Global Precipitation Measurement) IMERG (Integrated Multi-satellitE Retrievals) Final Precipitation Level 3 data was used to illustrate how well the proposed LNP model can produce the spatial distribution of a rainfall event. The data was downloaded from https://disc.gsfc.nasa.gov/datasets/GPM_3IMERGDF_06. The GPM data set is the gridded precipitation data produced by merging multiple satellite-based microwave data with calibration using ground truth data. The precipitation data by IDW with LNP using observed precipitation data and GPM are presented in Figure S9. The overall good agreement of dry area between two precipitation data sets was observed. However, the satellite-based precipitation data contains strong uncertainty^30–32^. The non-zero precipitation that appeared in observed precipitation on the exact location of weather stations are true value since these results were produced based on the ground truth. Thus, the satellite-based precipitation leads to false estimation on some stations. Nevertheless, the result supports that the LNP approach is useful and can be applied to calibrate microwave-based precipitation rate estimation.

## Summary and conclusions

When spatially interpolating a precipitation event, dry areas with zero precipitation have historically been ignored. Water management and impact assessments of climate change resulting from precipitation events require determining wet and dry regions appropriately. The current study proposed a novel approach to delineate wet and dry regions by assigning LNP to stations with precipitation values of zero. The proposed LNP method was tested with weather stations covering South Korea.

The overall results indicate that the LNP method can adequately delineate wet and dry regions with the appropriate tuning parameter. Additionally, the tuning parameter plays a special role in that its value increases with longer precipitation durations and denser networks. It is further suggested that a tuning parameter value of 0.5 – 1.5 can be used as a rule of thumb when the accuracy of the interpolation output is not critical.

The proposed LNP can be further tested over a much larger area, such as the United States, to enhance the spatial interpolation outputs. Additionally, the suggested LNP method can be employed for another variable, such as snow cover, for spatial interpolations over regions with sparse measurements. Also, the proposed LNP model cannot consider temporal dependency and physical features such as horizontal heterogeneity since it statistically interpolates the dry regions. Further model development can be made in the future study by taking the temporal dependency and physical features into account.

## Supplementary Information

Supplementary Information.
