# Supplementary material for: Latent negative precipitation for the delineation of a zero-precipitation area in spatial interpolations
Source: Sci Rep. 2021 Oct 14;11:20426. doi: 10.1038/s41598-021-99888-4 (PMC8516931; doi:10.1038/s41598-021-99888-4)
Supplement: Supplementary file 1 — Supplementary Information. [file 41598_2021_99888_MOESM1_ESM.docx]

Supporting Online Material for:

Latent negative precipitation for the delineation of a zero-precipitation area in spatial interpolations

Taesam Lee^1^ and Ju-young Shin^2^

^1^ Department of Civil Engineering, ERI, Gyeongsang National University,

501 Jinju-daero, Jinju, Gyeongnam, South Korea, 660-701

^2^High-impact Research Department, National Institute of Meteorological Sciences

, Jeju, South Korea

Supplementary Table

Table S 1. Locations of Employed Weather Stations

| No. | Lat. | Long. | No. | Lat. | Long. | No. | Lat. | Long. |
| --- | --- | --- | --- | --- | --- | --- | --- | --- |
| 1 | 38.25 | 128.56 | 26 | 35.56 | 129.32 | 51 | 36.27 | 126.92 |
| 2 | 38.15 | 127.30 | 27 | 35.17 | 128.57 | 52 | 36.11 | 127.48 |
| 3 | 37.90 | 127.06 | 28 | 35.17 | 126.89 | 53 | 35.73 | 126.72 |
| 4 | 37.89 | 126.77 | 29 | 35.10 | 129.03 | 54 | 35.61 | 127.29 |
| 5 | 37.68 | 128.72 | 30 | 34.85 | 128.44 | 55 | 35.56 | 126.87 |
| 6 | 37.90 | 127.74 | 31 | 34.82 | 126.38 | 56 | 35.40 | 127.40 |
| 7 | 37.75 | 128.89 | 32 | 34.74 | 127.74 | 57 | 35.66 | 127.52 |
| 8 | 37.51 | 129.12 | 33 | 34.69 | 125.45 | 58 | 34.69 | 126.92 |
| 9 | 37.57 | 126.97 | 34 | 34.40 | 126.70 | 59 | 34.55 | 126.57 |
| 10 | 37.48 | 126.62 | 35 | 34.47 | 126.32 | 60 | 34.62 | 127.28 |
| 11 | 37.34 | 127.95 | 36 | 33.51 | 126.53 | 61 | 36.94 | 128.91 |
| 12 | 37.27 | 126.99 | 37 | 33.29 | 126.16 | 62 | 36.87 | 128.52 |
| 13 | 37.18 | 128.46 | 38 | 33.39 | 126.88 | 63 | 36.63 | 128.15 |
| 14 | 36.97 | 127.95 | 39 | 33.25 | 126.57 | 64 | 36.53 | 129.41 |
| 15 | 36.78 | 126.49 | 40 | 35.16 | 128.04 | 65 | 36.36 | 128.69 |
| 16 | 36.99 | 129.41 | 41 | 37.71 | 126.45 | 66 | 36.13 | 128.32 |
| 17 | 36.64 | 127.44 | 42 | 37.49 | 127.49 | 67 | 35.98 | 128.95 |
| 18 | 36.37 | 127.37 | 43 | 37.26 | 127.48 | 68 | 35.67 | 127.91 |
| 19 | 36.22 | 127.99 | 44 | 38.06 | 128.17 | 69 | 35.57 | 128.17 |
| 20 | 36.57 | 128.71 | 45 | 37.68 | 127.88 | 70 | 35.49 | 128.74 |
| 21 | 36.41 | 128.16 | 46 | 37.17 | 128.99 | 71 | 35.41 | 127.88 |
| 22 | 36.03 | 129.38 | 47 | 37.16 | 128.19 | 72 | 34.89 | 128.60 |
| 23 | 36.01 | 126.76 | 48 | 36.49 | 127.73 | 73 | 34.82 | 127.93 |
| 24 | 35.88 | 128.65 | 49 | 36.78 | 127.12 |  |  |  |
| 25 | 35.84 | 127.12 | 50 | 36.33 | 126.56 |  |  |  |

Table S2 Kolmogorov-Smirnov test statistics of precipitation interpolated using IDW wo/w LNP to the observed precipitation.

| Duration (hr) | IDW without LNP | IDW with LNP |
| --- | --- | --- |
| 1 | 0.592 | 0.048 |
| 2 | 0.573 | 0.047 |
| 3 | 0.565 | 0.049 |
| 4 | 0.545 | 0.042 |
| 6 | 0.527 | 0.046 |
| 8 | 0.507 | 0.051 |
| 12 | 0.476 | 0.048 |
| 24 | 0.414 | 0.039 |

Supplementary Figure


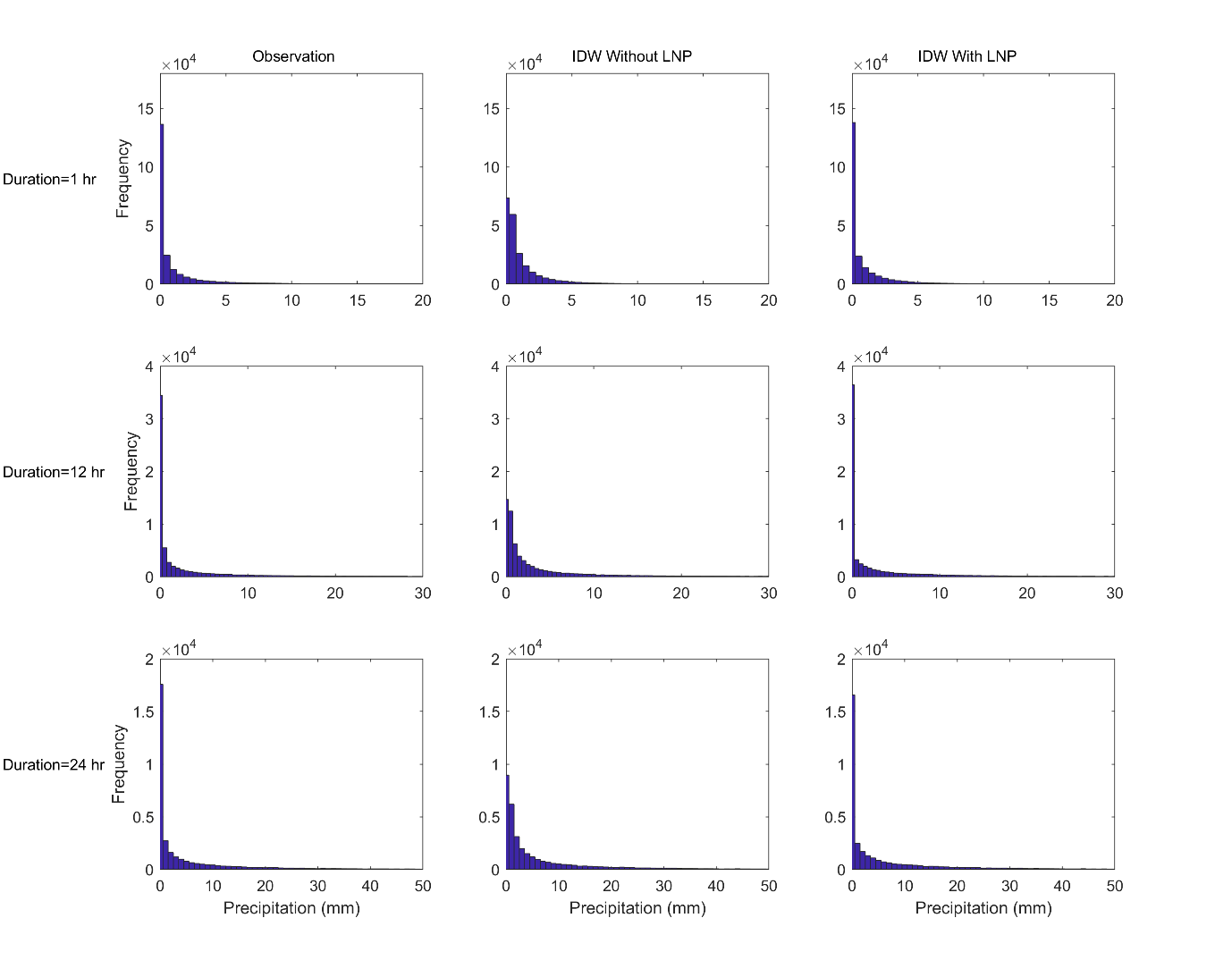


Figure S 1 Histograms of observed and interpolated precipitations using IDW wo/w LNP for 1-, 12-, and 24-hr across South Korea.


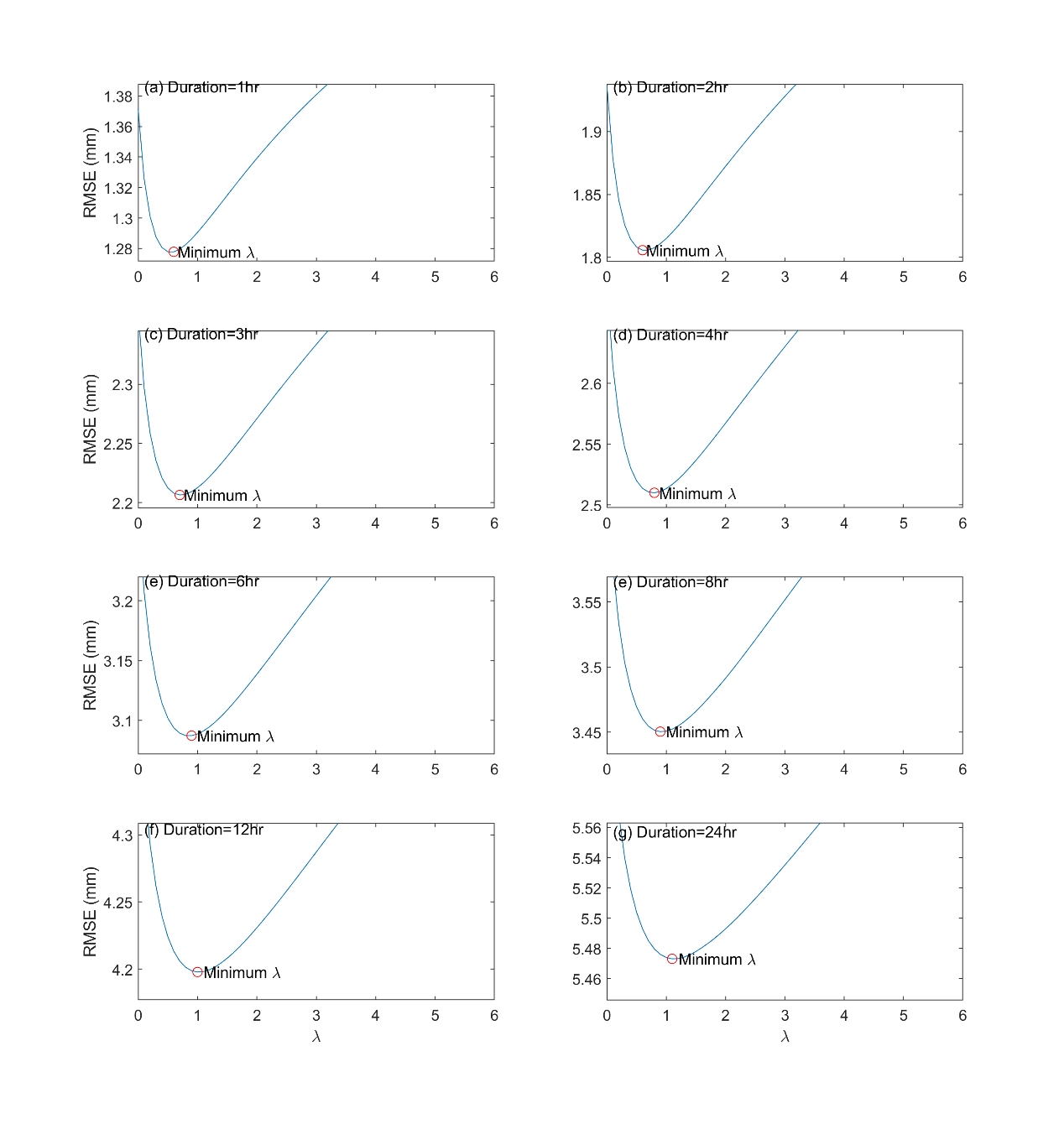


Figure S 2.Finding optimal lambda with different rainfall duration with 10 weather stations. Note that the average distance to the nearest station in the current study is 30km.


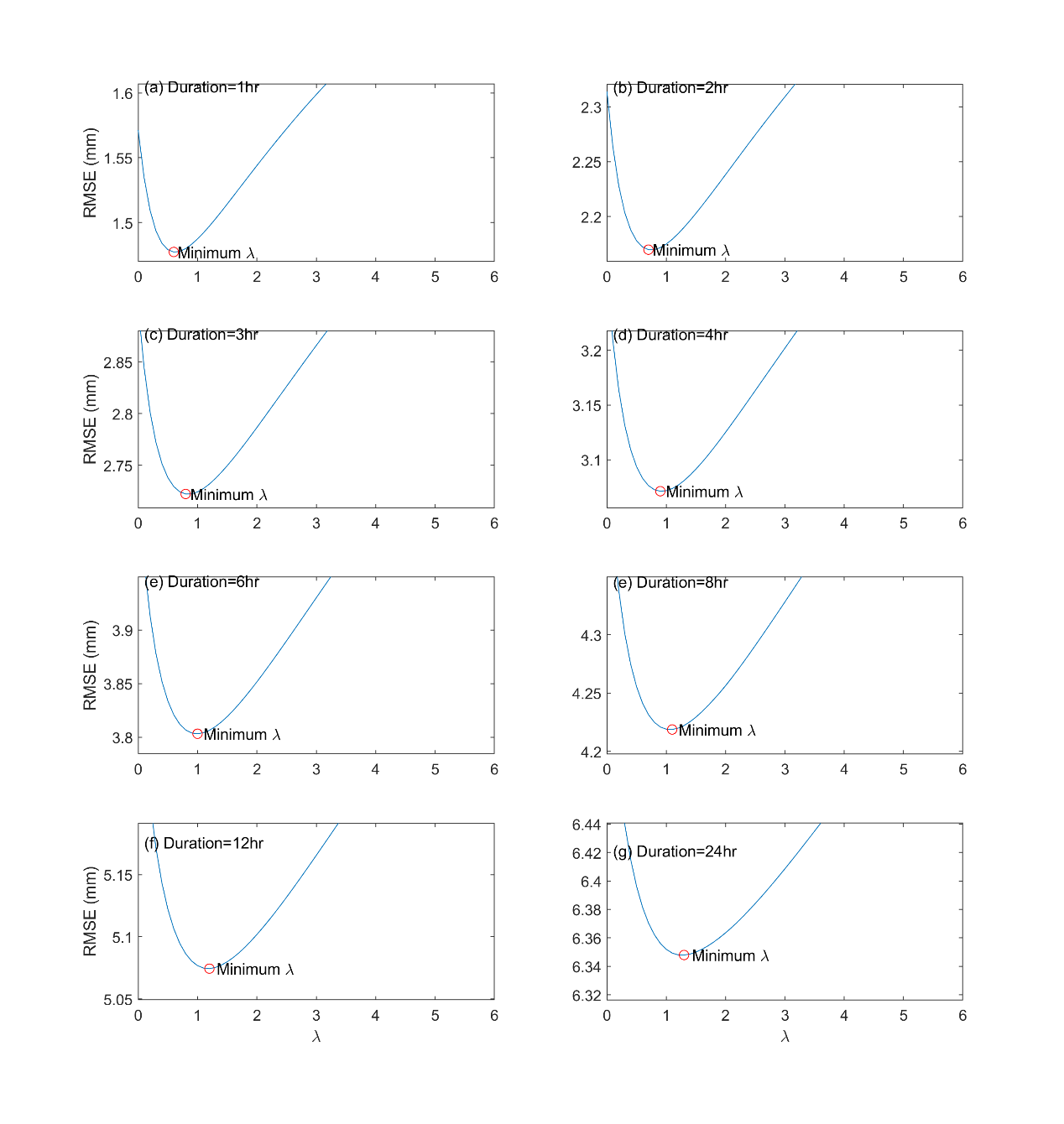


Figure S 3.Finding optimal lambda with different rainfall duration with 20 weather stations


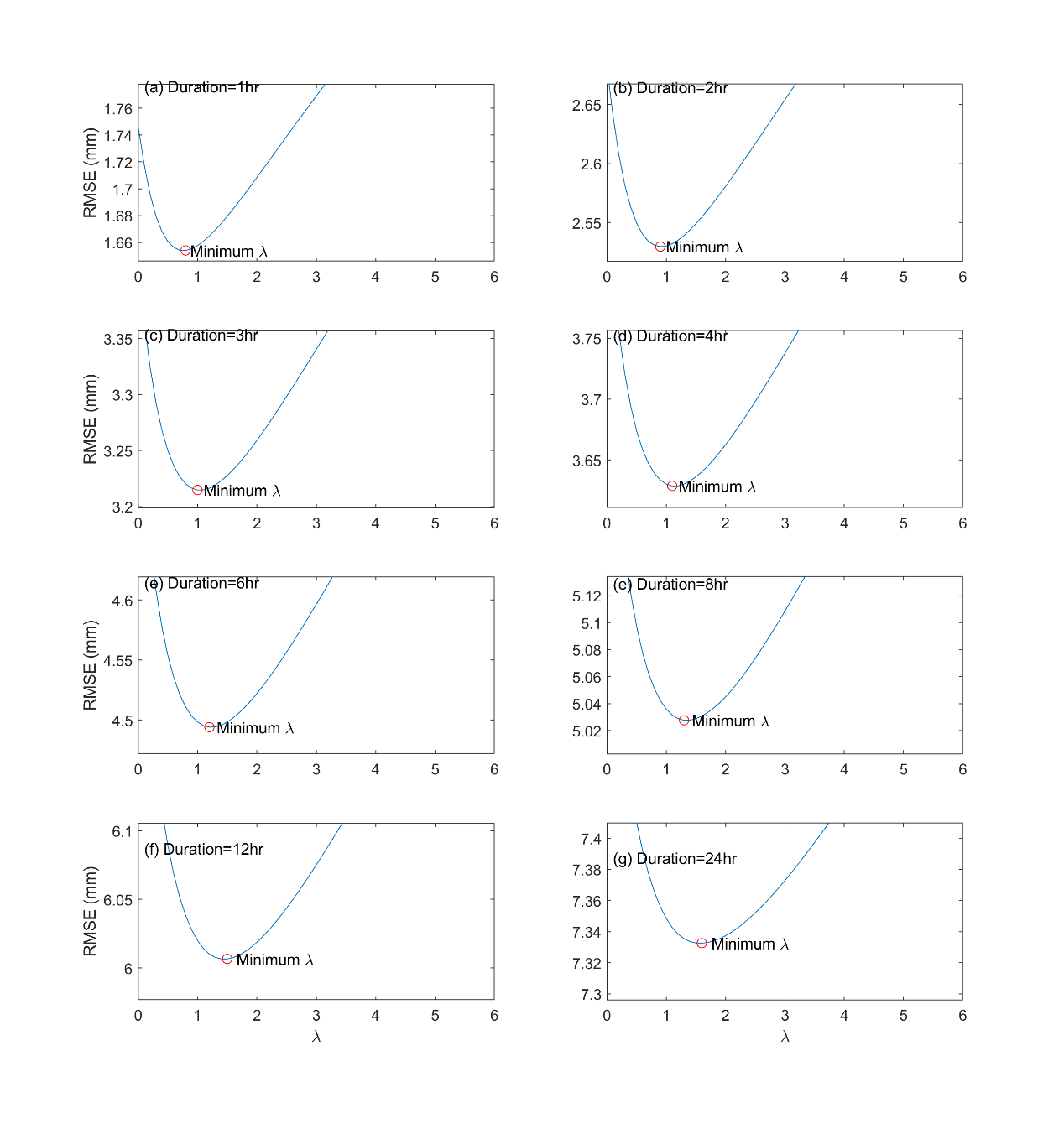


Figure S 4. Finding optimal lambda with different rainfall duration with 30 weather stations


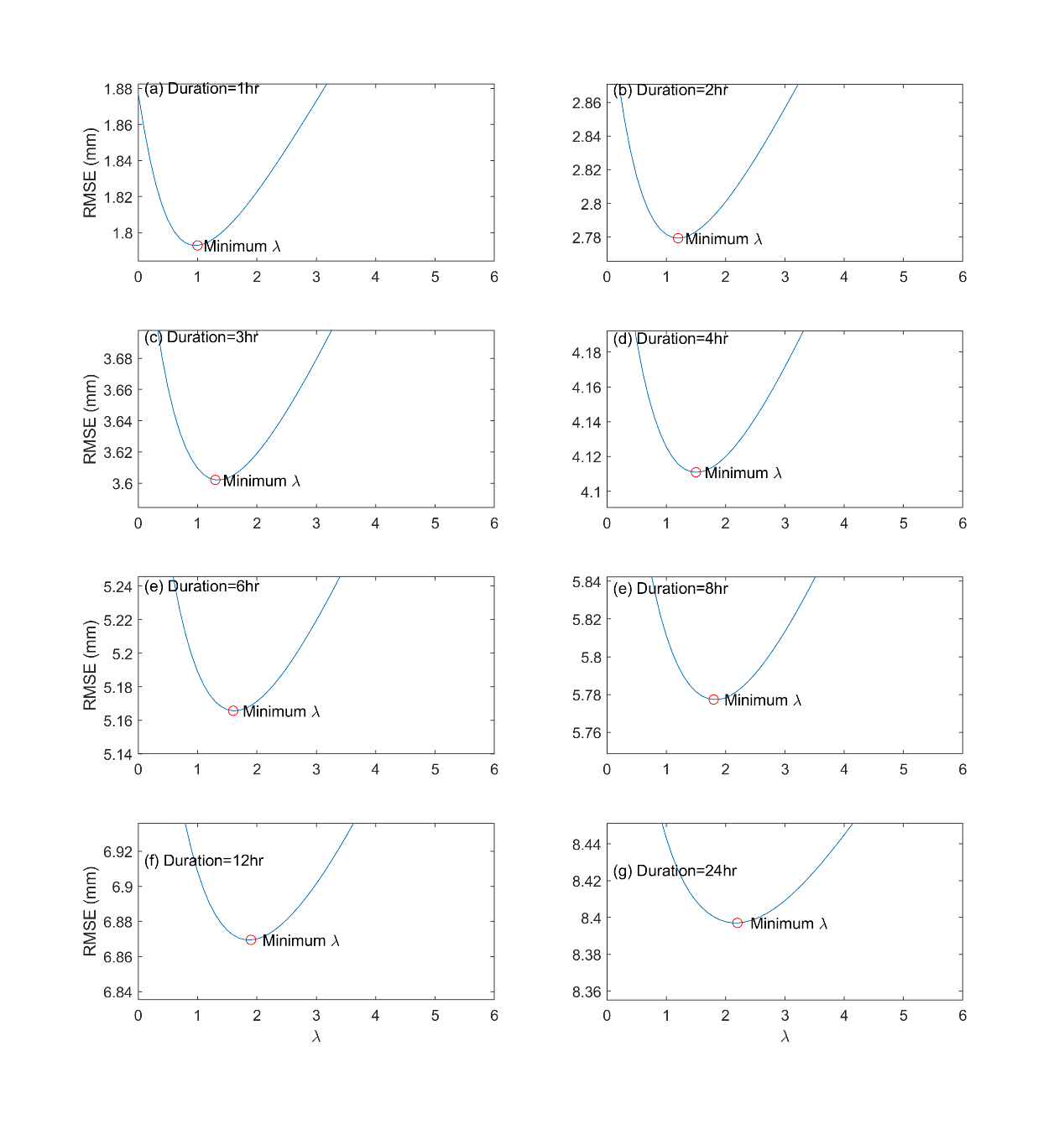


Figure S 5. Finding optimal lambda with different rainfall duration with 40 weather stations


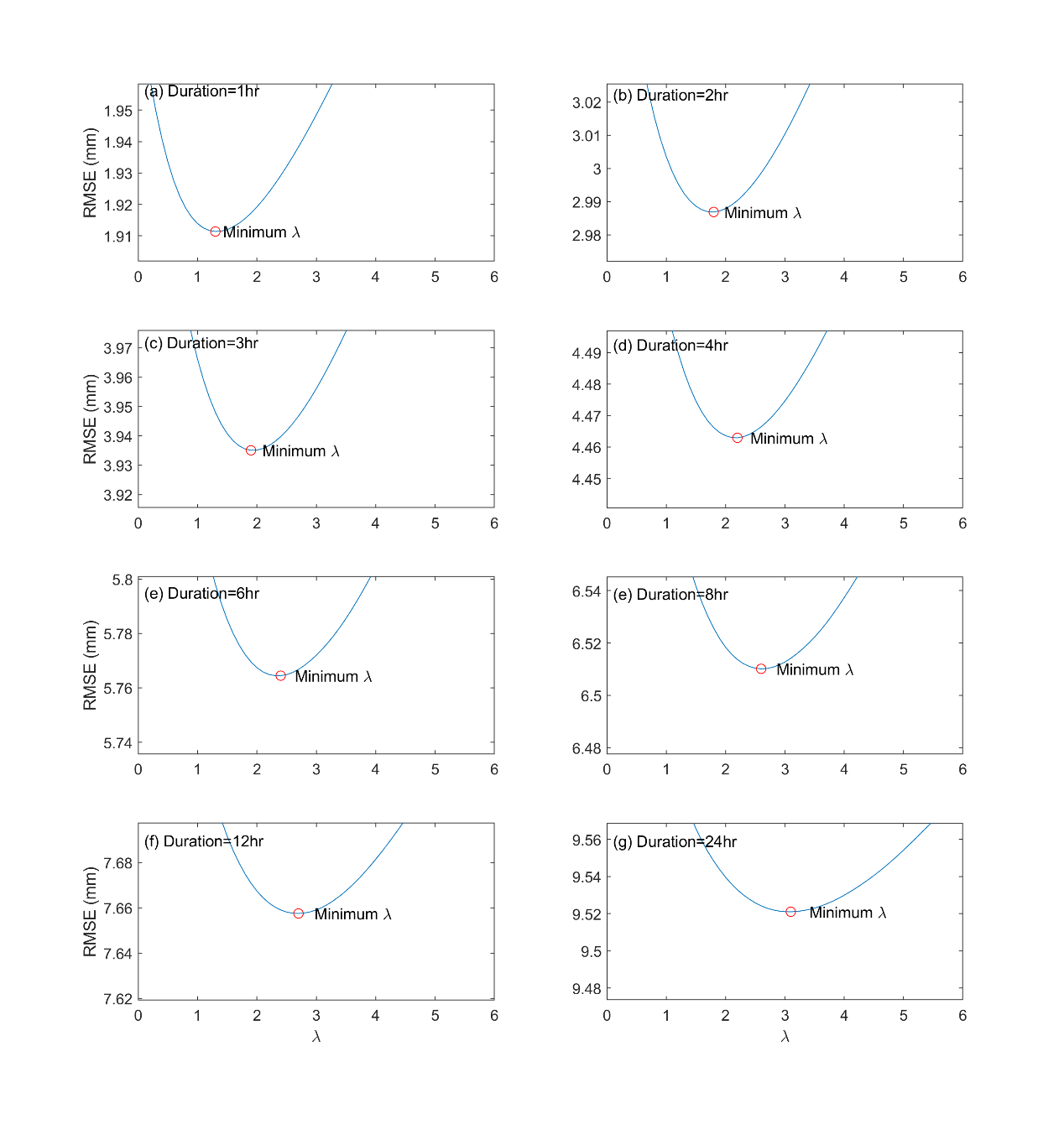


Figure S 6. Finding optimal lambda with different rainfall duration with 50 weather stations


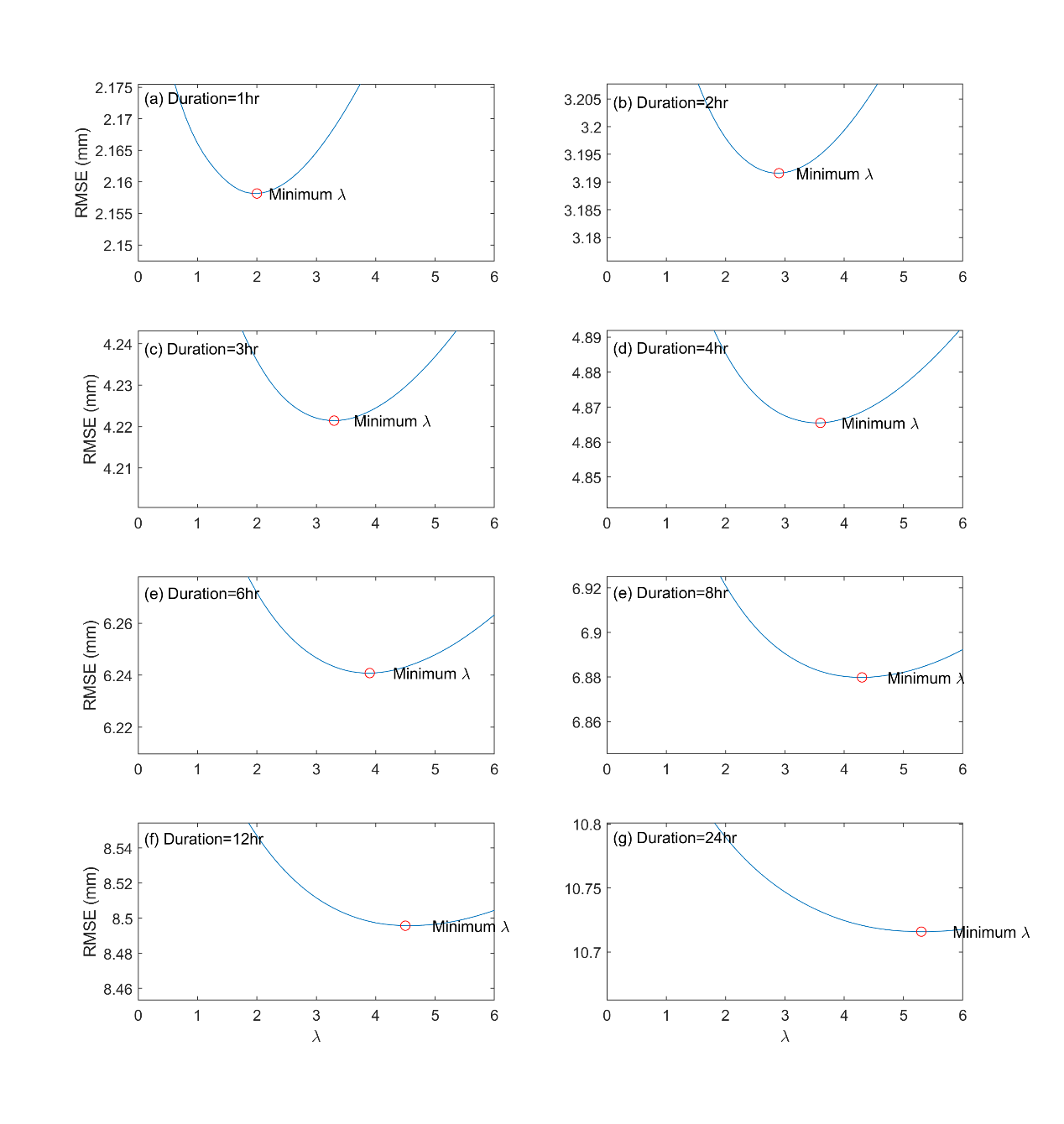


Figure S 7. Finding optimal lambda with different rainfall duration with 60 weather stations


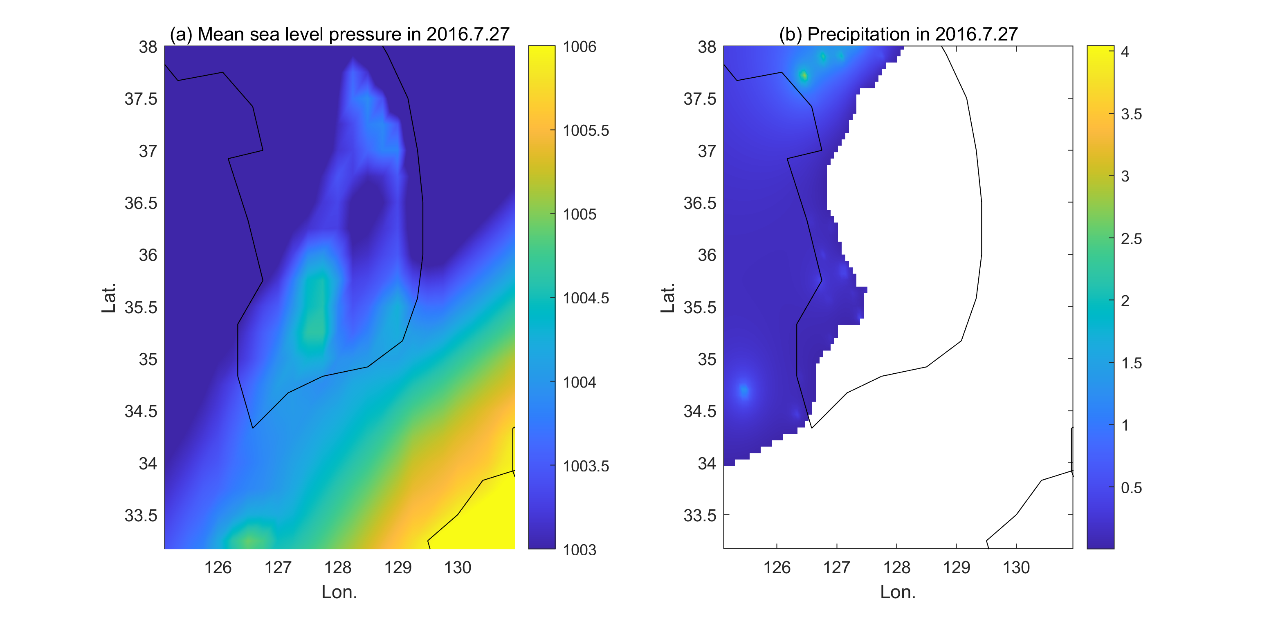


Figure S 8. Daily mean sea level pressure from ERA5 reanalysis data and daily precipitation interpolated using LNP in July 27 2016. Note that white colored cells indicate dry areas, i.e., no precipitation. The figures were created by MATLAB 2021a (https://www.mathworks.com/products/matlab.html).


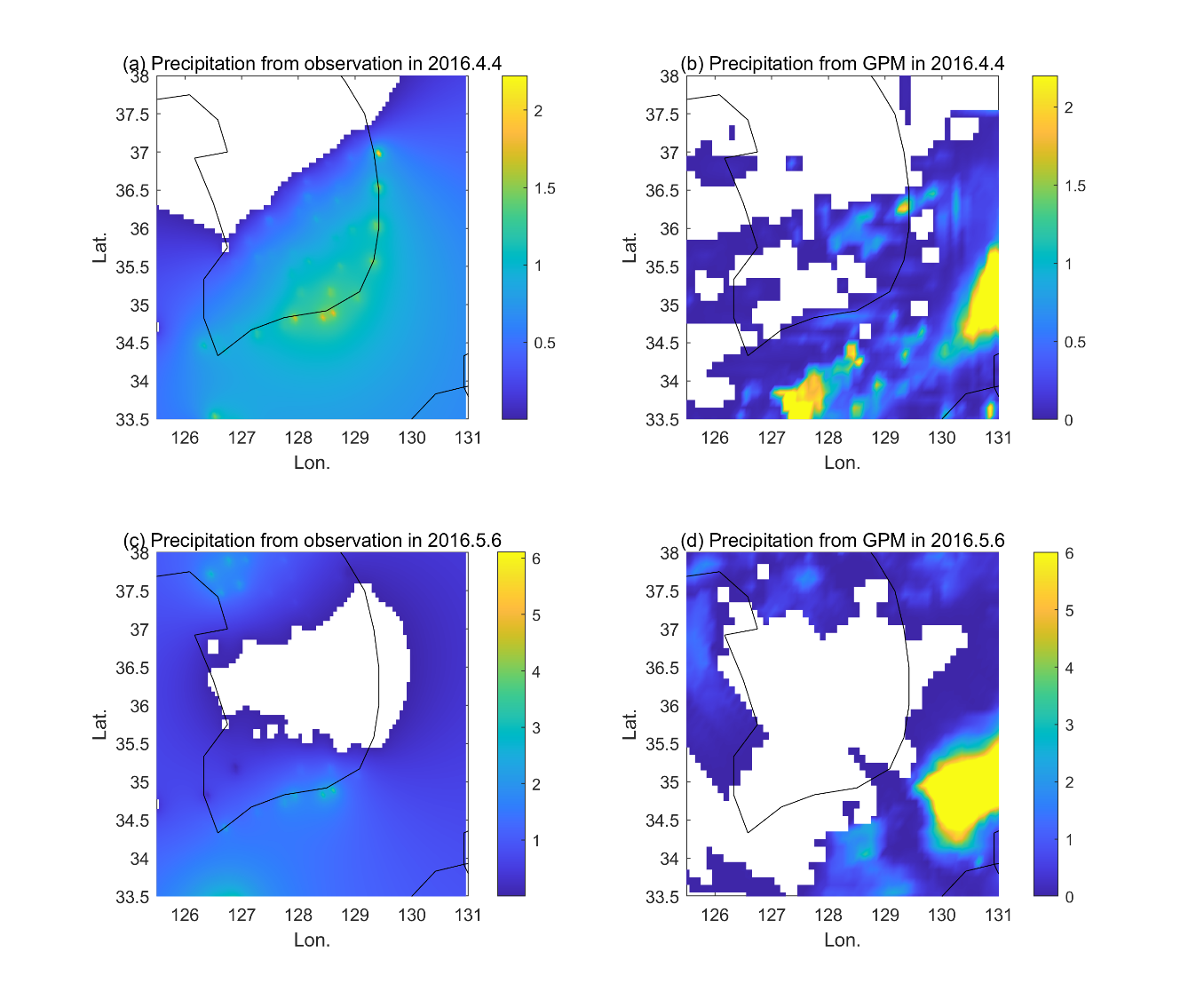


Figure S 9. Comparison of precipitation data from IDW with LNP and GPM. Note that white coloured cells indicate dry areas, i.e., no precipitation. The figures were created by MATLAB 2021a (https://www.mathworks.com/products/matlab.html).
